# PMCA Ca^2+^ clearance in dental enamel cells depends on the magnitude of cytosolic Ca^2+^

**DOI:** 10.1096/fj.202201291R

**Published:** 2023-01

**Authors:** Guilherme Henrique Souza Bomfim, Marta Giacomello, Rodrigo S. Lacruz

**Affiliations:** 1 Department of Molecular Pathobiology, New York University College of Dentistry, New York, New York, USA; 2 Department of Biology, University of Padova, Padua, Italy; 3 Department of Biomedical Sciences, University of Padova, Padua, Italy

**Keywords:** ameloblasts, Ca^2+^ clearance, Ca^2+^ signaling, enamel cells, PMCA

## Abstract

Enamel formation (amelogenesis) is a two-step process whereby crystals partially grow during the secretory stage followed by a significant growth expansion during the maturation stage concurrent with an increase in vectorial Ca^2+^ transport. This requires tight regulation of cytosolic Ca^2+^ (_c_Ca^2+^) concentration in the enamel forming ameloblasts by controlling Ca^2+^ influx (entry) and Ca^2+^ extrusion (clearance). Gene and protein expression studies suggest that the plasma membrane Ca^2+^-ATPases (PMCA1-4) are likely involved in _c_Ca^2+^ extrusion in ameloblasts, yet no functional analysis of these pumps has been reported nor whether their activity changes across amelogenesis. PMCAs have high Ca^2+^ affinity and low Ca^2+^ clearance which may be a limiting factor in their contribution to enamel formation as maturation stage ameloblasts handle high Ca^2+^ loads. We analyzed PMCA function in rat secretory and maturation ameloblasts by blocking or potentiating these pumps. Low/moderate elevations in _c_Ca^2+^ measured using the Ca^2+^ probe Fura-2-AM show that secretory ameloblasts clear Ca^2+^ faster than maturation stage cells through PMCAs. This process was completely inhibited by an external alkaline (pH 9.0) solution or was significantly delayed by the PMCA blockers vanadate and caloxin 1b1. Eliciting higher _c_Ca^2+^ transients via the activation of the ORAI1 Ca^2+^ channel showed that the PMCAs of maturation ameloblasts were more efficient. Inhibiting PMCAs decreased the rate of Ca^2+^ influx via ORAI1 but potentiation with forskolin had no effect. Our findings suggest that PMCAs are functional Ca^2+^ pumps during amelogenesis regulating _c_Ca^2+^ upon low and/or moderate Ca^2+^ stimulus in secretory stage, thus participating in amelogenesis.

## 1 | INTRODUCTION

In epithelial cells like the enamel forming ameloblasts, the cytosolic Ca^2+^ (_c_Ca^2+^) concentration is tightly regulated by the balance between Ca^2+^ influx (entry) and Ca^2+^ extrusion (clearance).^1,2^ These processes in ameloblasts are mediated by a sophisticated machinery that first controls the uptake of Ca^2+^ largely via the store-operated Ca^2+^ entry (SOCE) pathway^3–6^ formed by the stromal interacting proteins, STIM1/2,^7,8^ and the pore of the channel comprised of the ORAI1-3 proteins.^9,10^ The Ca^2+^ buffering and extrusion tool-kit in this context is less known but plasma membrane Ca^2+^ pumps and Ca^2+^ exchangers, Ca^2+^-binding proteins and buffering by intracellular organelles have been reported.^3,11–15^ Enamel is the most calcified vertebrate tissue, requiring a steady supply of Ca^2+^ during development (amelogenesis), a process commonly classified as the formative (secretory) and mineralization (maturation) stages.^16^ In ameloblasts, the general consensus is that Ca^2+^ is transported via a transcellular route before its extrusion into the enamel space at the apical pole of the cells.^2^ Ca^2+^ availability in ameloblasts is not only essential to mineralize the enamel hydroxyapatite crystals, but it also acts as a second messenger capable of regulating the expression of the enamel matrix protein.^17,18^

The removal of _c_Ca^2+^ in epithelial cells following a rise in its concentration is largely mediated by Ca^2+^ pumps and Ca^2+^ exchangers.^19–21^ Plasma membrane Ca^2+^-ATPases (PMCAs) are high affinity Ca^2+^ pumps but have low transport capacity, whereas the opposite is true for the Na^+^/Ca^2+^ exchangers (NCXs/NCKXs).^19^ This difference in their ability to dissipate _c_Ca^2+^ suggests that PMCAs are fine tuners of _c_Ca^2+^ operating at levels where the exchangers are less efficient.^19^ At least one study has reported functional data on NCX in ameloblasts^22^ but currently, there are no reports on whether the PMCAs are functional in ameloblasts. The present study aims to address the function of PMCAs in secretory and maturation stage ameloblasts as these two cell types play different roles during enamel formation,^3,16,23^ and show distinct Ca^2+^ handling capacity.^1,14,16,24,25^

PMCAs belong to the family of P-type ATPases acting as enzymatic pumps characterized by a temporary high-energy conservation of ATP in the form of a phosphorylated enzyme intermediate.^26,27^ They remove Ca^2+^ across the cell membrane against the electrochemical gradient with a stoichiometry of 1:1 for Ca^2+^/ATP, exchanging protons (H^+^) inward in the process.^28^ PMCAs have a low *Kd* for Ca^2+^: ~10–20 μM in a resting state, decreasing to ≤1 μM when activated.^29^ PMCAs are regulated by several processes and proteins: for example, they are activated by calmodulin, by protein kinases (PKA, PKC),^20,30,31^ and by cyclic AMP (cAMP).^32^ There are four gene variants of the PMCA coding genes (*ATP2B1-4*) with distinct tissue distribution.^27,29^ PMCA1 and 4 are ubiquitously expressed, while the tissue distribution of PMCA2 and PMCA3 is more restricted.^29^ Human pathologies linked to PMCA dysfunction are represented by hereditary deafness associated with loss of PMCA2 that is expressed in the organ of Corti.^33^ In mice, loss of PMCA2 causes a decrease in Ca^2+^ concentration in milk.^34^ Loss of both copies of PMCA1 caused embryonic lethality, but heterozygous mutants had no observable disease phenotype.^35,36^ By contrast, PMCA4 deletion did not cause pathology beyond infertility in male mice.^36^ These reports suggest that there is some compensation between PMCAs.

Protein and mRNA expression studies have shown that PMCA1, PMCA3, and PMCA4 are highly expressed in secretory stage ameloblasts^37^ yet their specific functional properties in regulating ameloblast Ca^2+^ homeostasis and/or mineralization remain unknown. Using rat primary enamel cells from the secretory and maturation stages, we show that upon induction of a small rise in _c_Ca^2+^ transients, secretory ameloblasts have higher rates of Ca^2+^ clearance capacity through PMCAs than maturation cells that can be inhibited by alkalizing (pH 9.0) the extracellular solution. The PMCA blockers vanadate and caloxin 1b1 substantially decreased Ca^2+^ clearance, especially in the secretory stage. We also show that PMCA function can be enhanced by the activation of PKA with forskolin or the activation of the second messenger cAMP (cyclic adenosine 3′,5′-monophosphate) using 8-Bromo-cAMP. Evoking a substantial elevation of _c_Ca^2+^ showed that maturation stage PMCAs likely acted in combination with the Ca^2+^ exchangers to clear intracellular Ca^2+^. Combined, our studies indicate that PMCA pumps are active in ameloblasts, being more effective in the secretory stage at regulating _c_Ca^2+^ after small increase in the intracellular Ca^2+^ levels.

## 2 | MATERIALS AND METHODS

### 2.1 | Animal use

All procedures employed in this study were conducted in accordance with guidelines approved by the Institutional Animal Care and Use Committee (IACUC) of New York University College of Dentistry (protocol # IA16-00625). Experiments were carried out in male and/or female (100 ± 10 g) Sprague Dawley rats ~5 weeks old. Animals were obtained from Charles River Laboratories (Wilmington, USA).

### 2.2 | Primary culture of enamel ameloblast cells

Secretory and maturation enamel organ cells were isolated from the rat lower incisors as we have described in detail elsewhere.^24,25,38^ We used the rat incisors because it is well-established system to study enamel development.^39^ Briefly, to obtain single ameloblast populations, isolated cell clumps from secretory and maturation stages were transferred to Eppendorf tubes containing 1 ml of Hanks’ balanced salt solution (Thermo Fisher, USA; #:14065-056) with 1% Antibiotic–Antimycotic (Thermo Fisher, USA; #:15240-062). Subsequently, secretory and maturation cells were digested with 0.25 mg/ml of Liberase (TL Roche, Germany; #414654) for 40 min at 37 °C in a 5%-CO_2_ incubator, manually pipetted (Gilson P1000L, USA; #:FA10006M) every 10 min to mechanically separate the cells. The enzymatic reaction was stopped by adding 2 ml of X-Vivo^™^ 15 (Lonza Bioscience, USA; #:04418Q) cell media containing 10% FBS (Thermo Fisher, USA; #:12483-020) and 1% Penicillin–Streptomycin (Thermo Fisher, USA; #:15140-122). Cells were centrifuged at 500g for 5 min, washed twice and plated on 25 mm boro–silicate cover glass (Fisher Scientific, USA; #:12545102P) coated with Corning^™^ Cell-Tak (Fisher Scientific, USA; #:CB40240). Cells were used within 24 h after dissection. The purity of the enamel ameloblasts culture was confirmed labeling the possible fibroblasts growing using a PE-conjugated monoclonal anti-rat CD90 antibody (Biolegend, USA; #:202523) and analyzing gene markers for secretory and maturation stage (Figure S1) as we have reported.^40^

### 2.3 | _c_Ca^2+^ measurements

_c_Ca^2+^ measurements were performed as previously we described.^4,25,38^ Briefly, cells were incubated for 1 h at room temperature with 1 μM of the ratiometric Ca^2+^ probe Fura-2-AM (Thermo Fisher, USA; #:F1221). The regular Ringer’s solution contained the following composition (in mM): 155.0 NaCl; 4.5 KCl; 2.5 CaCl_2_; 1.0 MgCl_2_; 10 D-glucose; and 10 HEPES, pH 7.4 adjusted with NaOH. In the nominally Ca^2+^-free Ringer’s solution, the CaCl_2_ was omitted, and the osmolarity maintained adding 3.5 MgCl_2_. All reagents were obtained from Sigma-Aldrich, USA. _c_Ca^2+^ transients of secretory and maturation cells were measured at room (24 ± 2°C) temperature using a polycarbonate 260 μl chamber (Harvard Bioscience Inc., USA; #:RC21BR) mounted on an inverted microscope coupled to a perfusion system electrically controlled. Fluorescence recordings were obtained using the Nikon Ti2-E Eclipse inverted light microscope, equipped with an objective (Nikon S Fluor × 20; numerical aperture: 0.75) and a digital SLR camera (DS-Qi2; Nikon, Japan) controlled by computer software (NIS Elements version 5.20.01, USA). Cells were continuously perfused by a six-way perfusion system (VC-8 valve controller) at 5–6 ml per minute with a common outlet 0.28-mm tube driven by controlled valves (Harvard Bioscience Inc., USA). The Ca^2+^ probe Fura-2-AM was excited alternatively at 340 and 380 nm using a Lambda LS xenon-arc lamp (Sutter Instrument, USA) and/or pE-340 Fura (Cool Led, USA). Emitted fluorescence was collected through a 510 nm emission filter. Fluorescence images were generated at 5 s intervals, normalized and the ratio values were calculated using Image J (1.53 J). The Fura-2 calcium imaging calibration kit (Thermo Fisher, USA; #:F6774) was used to estimate _c_Ca^2+^ concentration, as we have reported,^38^ according to the manufacturer’s specifications. Standard control buffer (background fluorescence), zero free-Ca^2+^ buffer (free-Ca^2+^), and 39 μM free-Ca^2+^ buffer (saturating Ca^2+^) were used to convert the emission ratio at 340/380 nm excitation to estimate the free _c_Ca^2+^.^41,42^

### 2.4 | Drugs and solutions used in the inhibition/activation of PMCA

To stimulate a rise in _c_Ca^2+^, we used ATP (3 μM, 10 μM, and 30 μM) (Abcam, USA; #: ab146525) in regular Ringer’s solution as well as in a Na^+^-free solution, using N-methyl-d-glucamine (NMDG) as a Na^+^ replacement, to prevent the involvement of NCX/NCKX exchangers.^42–44^ The PMCA pumps were blocked at or near reaching the intracellular Ca^2+^ peak by perfusing an alkaline solution (pH 9.0) as previously reported.^44,45^ In addition to ATP stimulation in low concentrations, we also elicited a small rise in _c_Ca^2+^ using thapsigargin (TG, 15 min, 2 μM—Sigma-Aldrich, USA; #:T9033) in regular Ringer’s solution and the PMCA inhibitors, vanadate (VAN, 20 min, 1 mM—Tocris, USA; #:2821),^46^ or caloxin 1b1 (CALX, 5 min, 100 μM—AnaSpec, USA; #:AS64236)^47^ in Ca^2+^-free Ringer’s solution. The concentrations of both PMCA inhibitors we used had been reported in the literature as being efficient in blocking PMCA.^46,48,49,50,51^ A larger increase in _c_Ca^2+^ was evoked by the activation of SOCE using thapsigargin (TG, 20 min, 2 μM—Sigma-Aldrich, USA; #:T9033) in Ca^2+^-free Ringer’s solution followed by a re-addition of 2.5 mM of extracellular Ca^2+^ in normal Ringer’s solution as we have reported.^38,52^ We then monitored Ca^2+^ clearance in the presence or absence of an alkaline (pH 9.0) solution, or vanadate, in regular Ringer’s solution. PMCA function was activated using the cell-permeable cAMP analog, 8-Bromoadenosine-3′,5′-cyclic monophosphate (8-Br-cAMP, 30 min, 100 μM—Tocris, USA; #1140), and forskolin (FSK, 10 min, 10 μM or 30 μM—Sigma-Aldrich, USA; #:F6886) as reported^53–56^ using a Ca^2+^-free Ringer’s solution.

### 2.5 | Real-time PCR

Total RNA was isolated using the RNeasy Micro Kit (Qiagen^®^), as indicated by the manufacturer, followed by reverse transcription using the iScript^™^ cDNA Synthesis Kit (Bio-Rad, USA). For qRT-PCR, we used the SsoAdvanced^™^ Universal SYBR^®^ Green Supermix (Bio-Rad, USA) and performed the experiments in a CFX Connect thermocycler (Bio-Rad, USA). β-actin was used as the housekeeping gene. Relative quantification of the PMCA coding genes of isoform 1(*Atp2b1*), isoform 3 (*Atp2b3*), isoform 4 (*atp2b4*), PKA-α (*Prkaca*), PKA-β (*Prkacb*), PKC-α (*Prkca*), and PKC-γ (*Prkcg*), and the enamel gene markers *Odam* and *Enam* were determined by the 2^−ΔΔCT^ (delta delta CT) method expressing values relative to the secretory ameloblasts. All primers were used at 0.25 nM, and the forward and reverse sequences are shown in Table S1.

### 2.6 | Data analyses and statistics

All data, mathematical analyses, and graphs were analyzed and/or generated using the GraphPad Prism software version 9.4.1 (Inc., California, USA), as we previously described.^38,52,57^ Basal _c_Ca^2+^ levels were calculated averaging the values from 0 s to 60 s of each independent experiments. The Ca^2+^ clearance through PMCAs was analyzed measuring the Ca^2+^ efflux (clearance) rate parameter calculated in each individual trace and fitted by the nonlinear regression curve. The equation of this model has the following functions: Y = IF (X < X0, Y0, Plateau + (Y0-Plateau) * exp(−K*(X−X0))). Data represent the mean ± SEM of the minimum of three independent experiments. The total number of cells used in each set of experiment is indicated in the graphs. Differences between the means of the group data, that fit a normal distribution, were analyzed using one-way ANOVA followed by Tukey’s multiple comparisons post-hoc test or two-tailed unpaired Student’s *t* test. The limit of significance was established *p < .05; **p < .01; ***p < .001; ^#^*p* < .05; ^&^*p* < .05; ^@^*p* < .05 and n.s., non-significant, as indicated in each figure legend.

## 3 | RESULTS

### 3.1 | PMCA function in ameloblasts is inhibited by alkaline extracellular pH

Primary ameloblast cultures showed low fibroblast contamination (~5%–8%) (Figure S1). These contaminating cells were excluded from the analysis. qRT-PCR analysis validated the separation of secretory and maturation cell populations using stage-specific gene markers (Figure S1).

**FIGURE 1:**
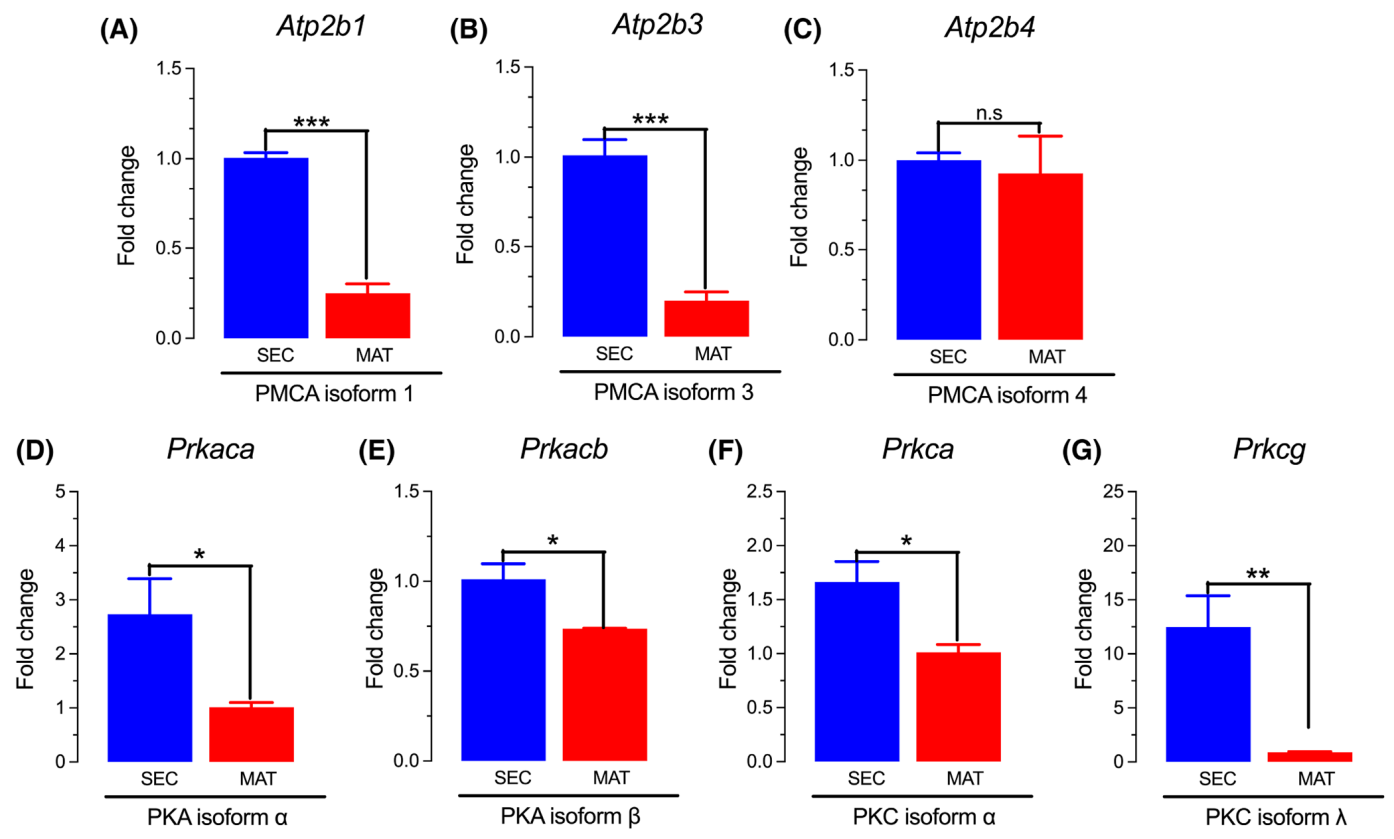
Gene expression of PMCA, PKA, and PKC coding genes in ameloblasts. Gene expression analysis quantified by qRT-PCR of the genes encoding PMCA1 (*ATP2B1*) (A), PMCA 3 (*ATP2B3*) (B) and PMCA4 (*ATP2B4*) (C), protein kinases (PKA) PKA-α (*Prkaca*) (D), PKA-β (*Prkacb*) (E), PKC-α (*Prkca*) (F), and PKC-γ (*Prkcg*) (G) in secretory (SEC) and maturation (MAT) stage ameloblasts. Data represent the mean ± SEM of 3–4 independent experiments analyzed by two-tailed unpaired Studen’s *t* test at *p < .05, **p < .01, ***p < .001. Fold changes were calculated relative to values in secretory ameloblasts, n.s., non-significant.

**FIGURE 2:**
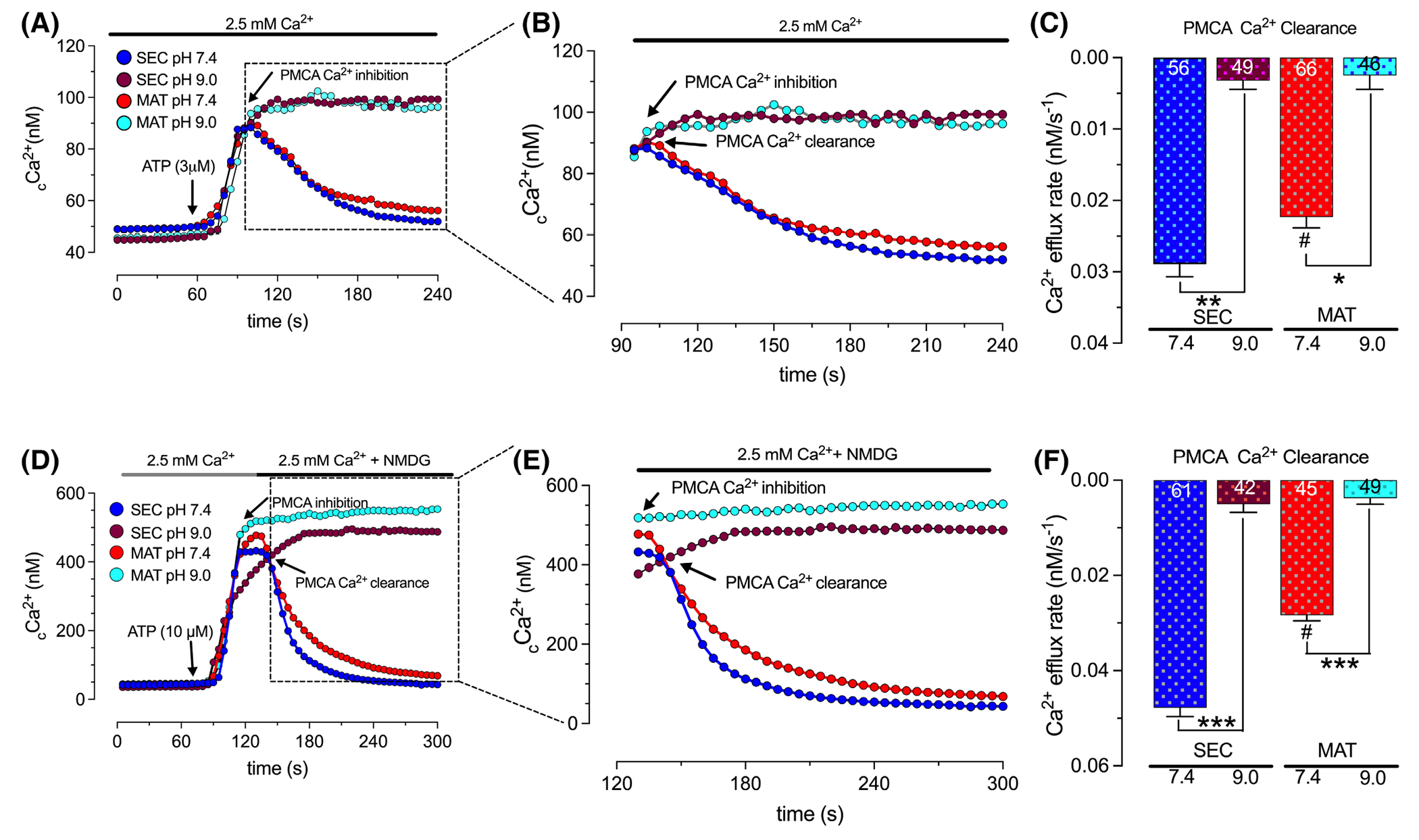
Secretory stage ameloblasts show higher Ca^2+^ clearance via PMCAs. Representative original traces of secretory (SEC) and maturation (MAT) ameloblasts showing _c_Ca^2+^ transients elicited by ATP 3 μM (A–C) or ATP 10 μM (D-F) in regular Ringer’s solution (pH 7.4) or in an alkaline (pH 9.0) solution. Ca^2+^ clearance was monitored after stimulation of _c_Ca^2+^ elevation by ATP. Experiments were performed in the absence (A-C) or presence of NMDG (D-F) in regular Ringer’s solution. Data represent the mean ± SEM from three to five independent experiments analyzed by one-way ANOVA followed by Tukey’s multiple comparison post-hoc test. Number of cells used per condition are included in the histograms. ***p < .001, **p < .01, or *p < .05 versus; #*p* < .05 (# denotes differences between SEC and MAT).

We next determined the expression of PMCA genes (*Atp2b1,3,4*) in secretory and maturation stage ameloblasts showing that PMCA1 and PMCA3 are the most abundant transcripts, particularly in the secretory stage (Figure 1A,B), confirming previous expression studies.^37^ Because PKA and PKC are known to activate PMCA, we also analyzed the expression profiles of these kinases.^30,31^ We show that the expression of the both *PKA* and *PKC* was higher in secretory ameloblasts (Figure 1D–G), consistent with the expression profile of PMCAs. To address the functional role of PMCA in enamel cells, we stimulated secretory and maturation stage ameloblasts with several low and/or moderate concentrations of the agonist ATP to elevate _c_Ca^2+^ because we have shown previously that the transient application of ATP increases _c_Ca^2+^ in ameloblasts, likely via the activation of the purinergic receptors P2RY1 or P2RY6.^25^ We show that ATP-mediated elevations of _c_Ca^2+^ is dose dependent (Figure S2). To address PMCA function in ameloblasts, we first stimulated the cells with a low ATP concentration (3 μM) which evoked a small elevation in _c_Ca^2+^ similar in secretory and maturation stage ameloblasts (Figure 2A). Measurements of the rate of Ca^2+^ clearance revealed that secretory ameloblasts extruded Ca^2+^ more rapidly than maturation stage ameloblasts (Figure 2B,C). To address if Ca^2+^ removal was PMCA mediated, we performed the same experiment perfusing the cells with an alkaline (pH 9.0) solution. Alkalinization prevents the H^+^ exchange required for PMCA function.^44,58^ We show that alkalinization entirely blocks Ca^2+^ clearance in both secretory and maturation stage cells (Figure 2A–C). Using a higher ATP stimulus (10 μM) in the presence of NMDG (without Na^+^) to prevent the activity of the Na^+^/Ca^2+^ exchangers (NCXs/NCKXs)^42–44^ resulted in similar _c_Ca^2+^ peaks in secretory and maturation, albeit these were higher in magnitude compared to the previous experiment (Figure 2A–C). As before, _c_Ca^2+^ was more rapidly removed in secretory ameloblasts (Figure 2D–F). The addition of NMDG did not show changes in the kinetics of Ca^2+^ clearance. However, alkalinizing the perfusate at or near the _c_Ca^2+^ peaks completely blocked Ca^2+^clearance suggesting that this was mediated by PMCA (Figure 2A–F). These results suggest that at this low level of _c_Ca^2+^ stimulus, the Na^+^/Ca^2+^ exchangers (NCXs/NCKXs) are not extensively involved in Ca^2+^ extrusion.

### 3.2 | Pharmacological inhibition of PMCA decreased Ca^2+^ clearance in ameloblasts

**FIGURE 3:**
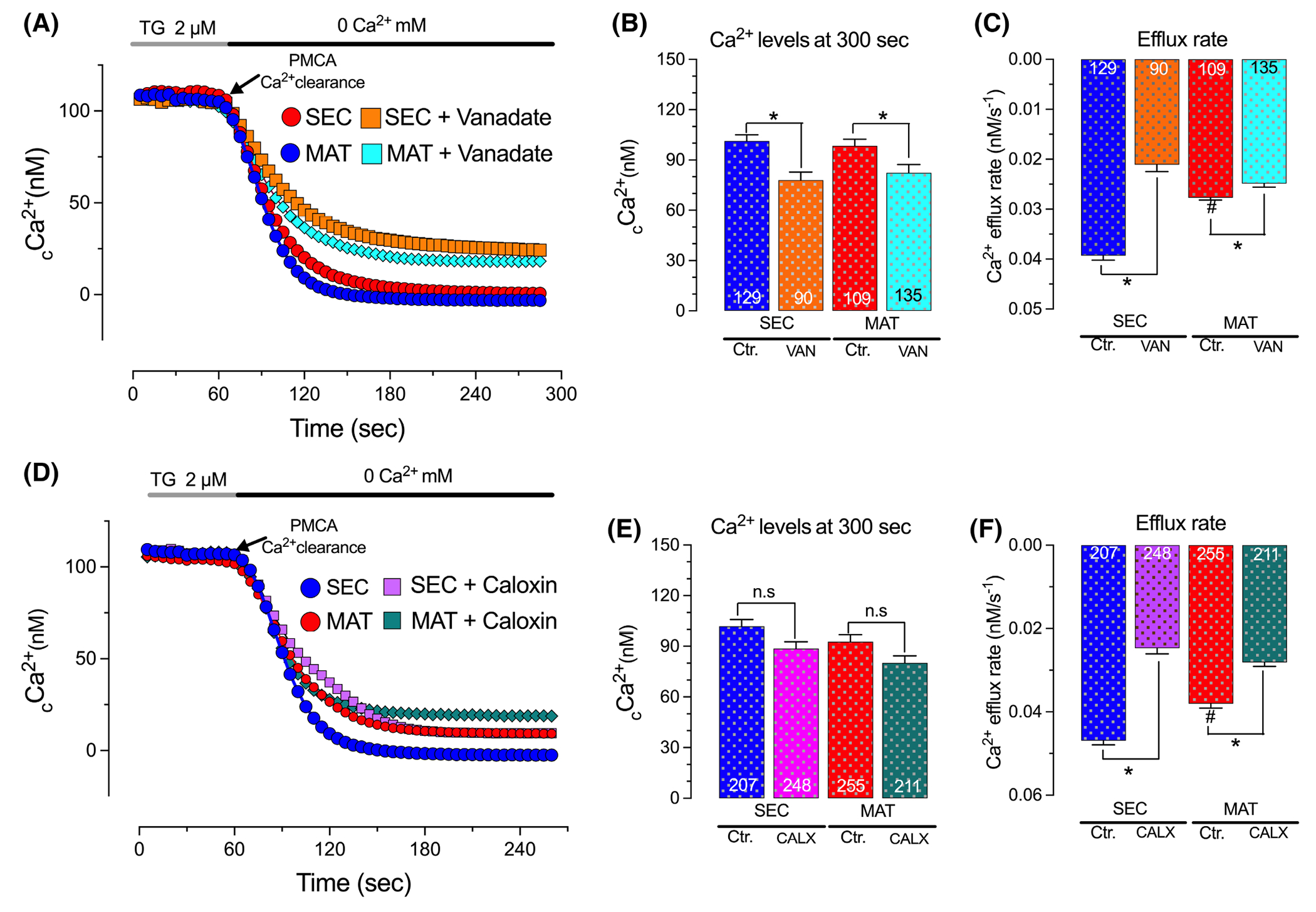
Pharmacological inhibition of PMCAs reduced Ca^2+^ clearance in enamel cells. Representative original traces of secretory (SEC) and maturation (MAT) ameloblasts showing _c_Ca^2+^ transients recorded after preincubation with thapsigargin (TG, 15 min, 2 μM) followed by perfusion of Ca^2+^-free Ringer’s (60 s to 270–300 s) (A and D). Ca^2+^ clearance via PMCAs was monitored in the presence/absence of vanadate (VAN, 20 min, 1 mM) or caloxin 1b1 (CALX, 5 min, 100 μM). Quantification of the rate of Ca^2+^ efflux (C and F) and Ca^2+^ levels at 270–300 s (B and E). Data represent the mean ± SEM of ≥90 cells from four to seven independent experiments analyzed by one-way ANOVA followed by Tukey’s multiple comparison post-hoc test. Number of cells used per condition are included in the histograms. *p < .05 versus ^#^*p* < .05 (# denotes differences between SEC-blue- and MAT-red).

As the previous experiments relied on preventing PMCAs Ca^2+^ clearance by extracellular alkalinization (pH 9.0), we performed additional experiments in the presence of vanadate and caloxin 1b1, which have been used to inhibit PMCAs.^47,50,59^ To evoke a modest rise in _c_Ca^2+^ we used the ER resident Ca^2+^-ATPase blocker, thapsigargin (2 μM). Next, we perfused the cells with a Ringer’s solution without Ca^2+^ in the presence of vanadate, which blocks PMCA at the E_2_ state from the internal side of the pump,^59^ and monitored Ca^2+^ clearance. We show that vanadate does not alter basal _c_Ca^2+^ but significantly reduced Ca^2+^ extrusion in both secretory and maturation stage ameloblasts, albeit the magnitude of this inhibition is greater in secretory stage ameloblasts (42% in secretory vs. 21% decrease in maturation, Figure 3A–C). Similarly, using caloxin 1b1, which has been described either as a PMCA blocker or has shown specificity for PMCA4, Ca^2+^ clearance is delayed in both secretory and maturation with a higher inhibition in the secretory ameloblasts (47% in secretory vs. 28% decrease in maturation, Figure 3D–F). These data are consistent with our previous observations using an alkaline solution to inhibit PMCAs highlighting a more prominent role of PMCA in secretory stage.

### 3.3 | PMCA inhibition affects Ca^2+^ extrusion and Ca^2+^ influx after stimulating SOCE

**FIGURE 4:**
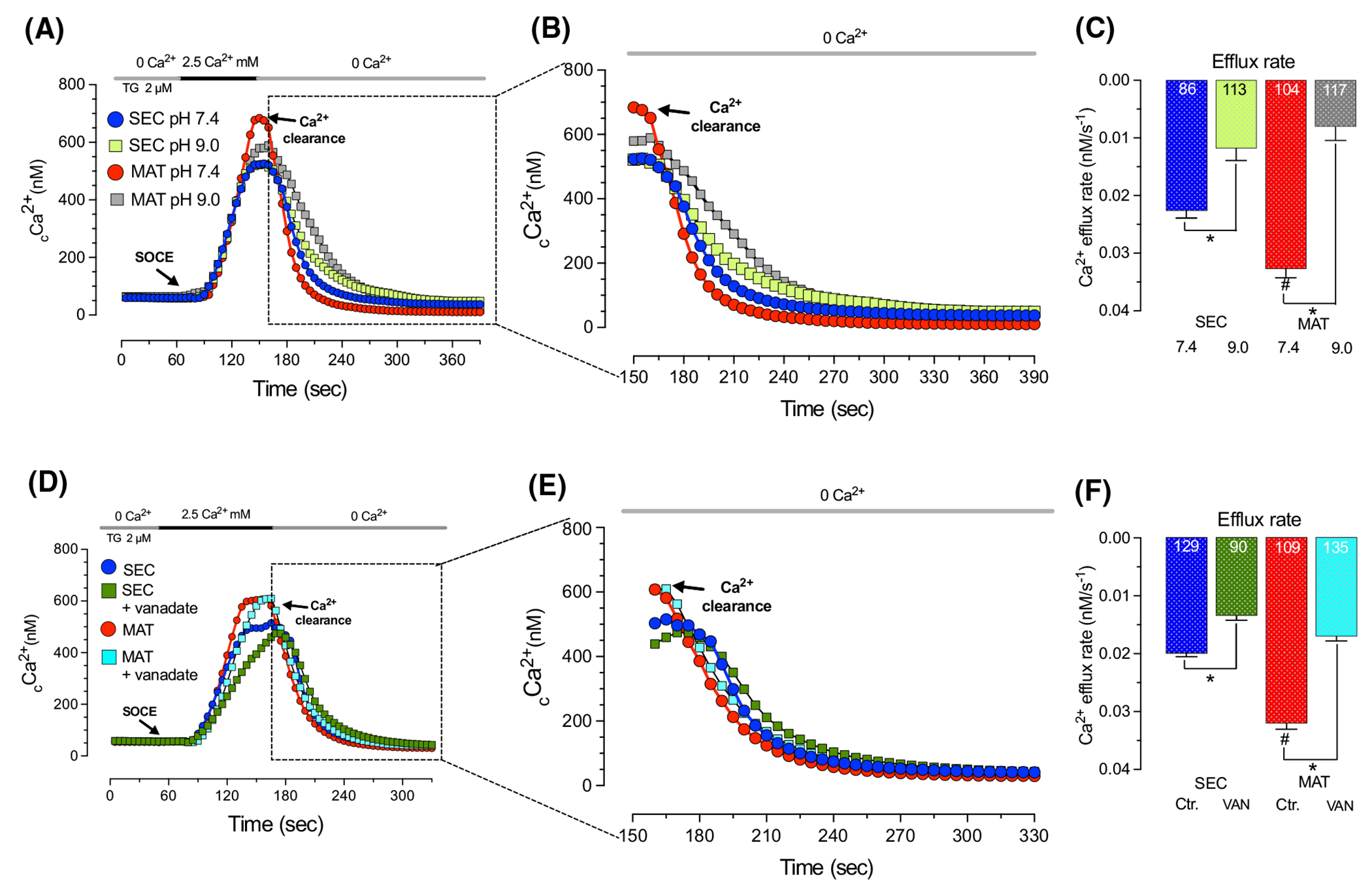
PMCA inhibition reduced Ca^2+^ clearance after SOCE stimulation in enamel cells. Representative original traces of secretory (SEC) and maturation (MAT) ameloblasts showing _c_Ca^2+^ transients elicited by SOCE recorded after preincubation with thapsigargin (TG, 20 min, 2 μM) followed by perfusion of 2.5 Ca^2+^ mM Ringer’s solution (A and D). Ca^2+^ clearance via PMCAs was monitored in the presence and absence of an alkaline (pH 9.0) solution (B) or vanadate (VAN, 20 min, 1 mM) (E). Quantification of the rate of Ca^2+^ efflux (clearance) (C and F). Data represent the mean ± SEM of ≥86 cells from three to five independent experiments analyzed by one-way ANOVA followed by Tukey’s multiple comparison post-hoc test. Number of cells used per condition are included in the histograms. *p < .05 versus ^#^*p* < .05 (# denotes differences between SEC-blue- and MAT-red-).

As discussed above, the main Ca^2+^ influx pathway in ameloblasts is SOCE.^6,25,38,60,61^ SOCE activation in ameloblasts elicits a robust increase in _c_Ca^2+^ being significantly higher in maturation stage ameloblasts.^4,25,38^ These levels may exceed the Ca^2+^ extrusion capacity of PMCAs but this has not been tested in ameloblasts to date. In addition, a previous report indicated that PMCA4 overexpression in Jurkat-T cells increased the effects of SOCE on downstream pathways such as the activation of NFAT,^62^ suggesting a link between Ca^2+^ influx via SOCE and PMCA-mediated Ca^2+^ efflux, which may also be important in enamel cells. To address these possibilities, we first stimulated SOCE in secretory and maturation stage ameloblasts using thapsigargin as we have reported^4,25,61^ before blocking PMCAs with an alkaline solution (pH 9.0). First, we addressed whether switching from a solution without Ca^2+^ to a solution containing 2.5 mM Ca^2+^ had an effect in _c_Ca^2+^. Monitoring Fura-2 AM signals during this procedure showed no significant alterations in intracellular Ca^2+^ (Figure S3). We then compared changes in _c_Ca^2+^ evoked by ATP and SOCE showing that activation of the latter evoked a greater increase in _c_Ca^2+^ (Figure S2). We next stimulated SOCE in the presence/absence of an alkaline solution perfused at the peak of the _c_Ca^2+^ transients, and we show that this delays the rate of Ca^2+^ clearance in both cell types but does not abolish Ca^2+^ extrusion (Figure 4A–C). Interestingly, at this level of _c_Ca^2+^, the delay in the rate of Ca^2+^ clearance is more pronounced in maturation (~74%) compared to secretory cells (~45%). To further analyze the effects of PMCA on SOCE and Ca^2+^ extrusion, we pretreated secretory and maturation stage ameloblasts with vanadate (1 mM, 20 min). In the presence of vanadate, the rate of Ca^2+^ uptake via SOCE was significantly reduced in secretory ameloblasts but not in maturation stage cells, indicating that in secretory cells, Ca^2+^ influx via SOCE is optimized when there is a participation of PMCA (Figure 4D). Ca^2+^ clearance was also delayed in both cell types in the presence of vanadate (Figure 4D–F). Combined, these data indicate that at high Ca^2+^ elevations induced by the activation of SOCE, PMCA only partially participate in Ca^2+^ extrusion, but affects SOCE mediated Ca^2+^ uptake in secretory cells.

### 3.4 | Potentiating PMCA function enhances Ca^2+^ extrusion in ameloblasts but not SOCE

**FIGURE 5:**
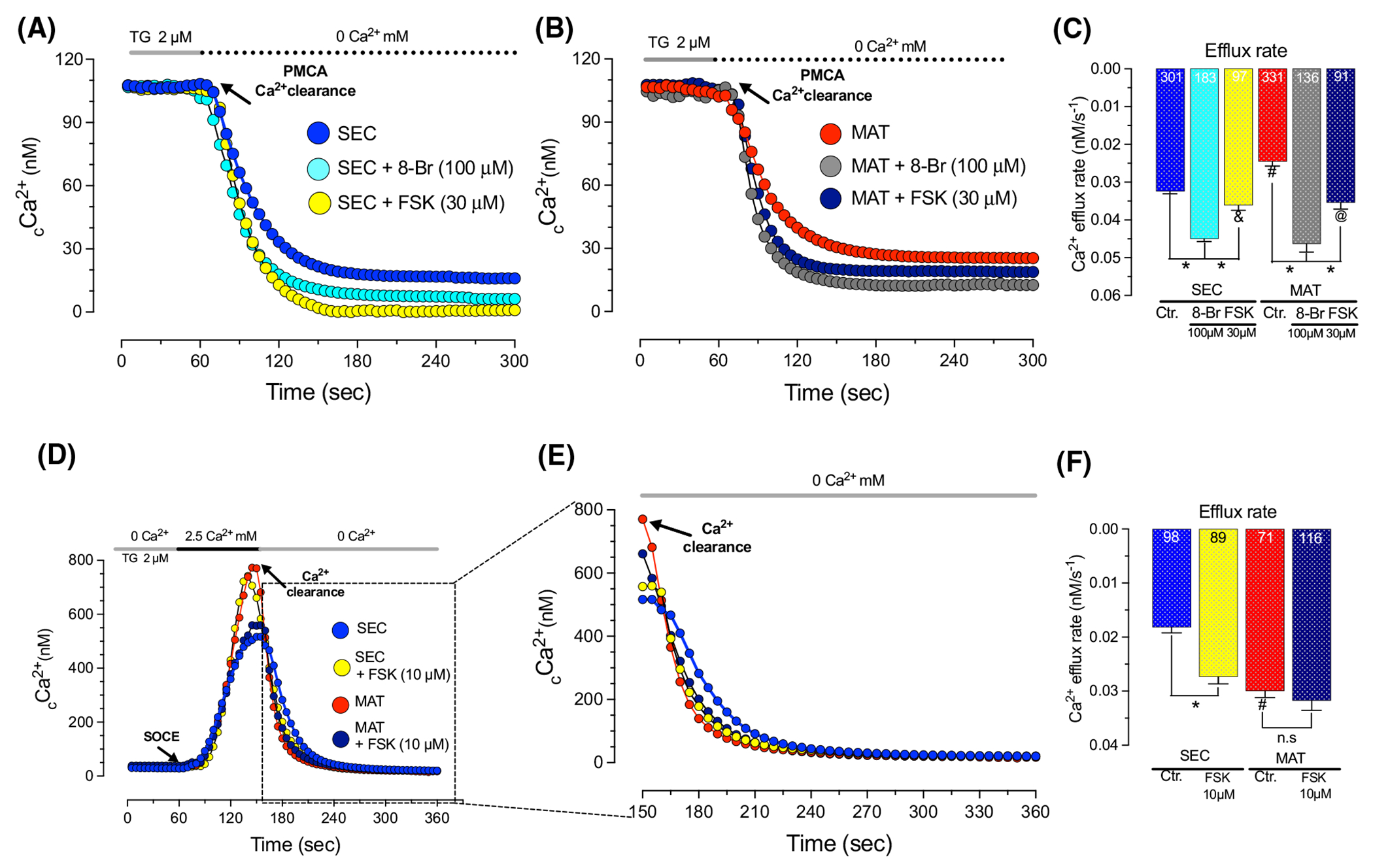
Forskolin and 8-Br-cAMP potentiate PMCA Ca^2+^ clearance in enamel cells. Representative original traces of secretory (SEC) and maturation (MAT) ameloblasts showing _c_Ca^2+^ transients recorded after preincubation with thapsigargin (TG, 15 min, 2 μM) followed by perfusion of Ca^2+^-free Ringer’s (60 to 300 s). Ca^2+^ clearance via PMCAs was monitored in the absence or presence of 8-Br-cAMP (100 μM, for 30 min) or forskolin (30 μM, for 10 min) (A and B). SOCE was recorded after a preincubation with thapsigargin (TG, 15 min, 2 μM) followed by perfusion of 2.5 Ca^2+^ mM Ringer’s solution in the absence or presence of FSK (10 μM, 10 min) (D and E). Quantification of the rate of Ca^2+^ efflux is shown in (C and F). Data represent the mean ± SEM of ≥71 cells from three to five independent experiments analyzed by one-way ANOVA followed by Tukey’s multiple comparison post-hoc test. Number of cells used per condition are included in the histograms. *p < .05 versus ^#^*p* < .05 (# denotes differences between SEC-blue- and MAT-red-); ^&^*p* < .05, (& denotes differences between SEC untreated-blue- and FSK treated-yellow), ^@^*p* < .05 (@ denotes differences between MAT untreated-red- and FSK treated-dark blue-), n.s. non-significant.

Elevated levels of cAMP activate PKA leading to the phosphorylation of PMCA increasing the activity of the Ca^2+^ pump.^27,32,63^ Forskolin is widely used to stimulate an increase in the levels of cAMP (via adenyl cyclase) thus activating the PKA pathway,^54,64^ similar to the effects of 8-Br-cAMP.^65^ To determine if PMCA function can be enhanced in enamel cells, we elicited a small elevation of _c_Ca^2+^ using thapsigargin (2 μM), and then treated the cells with forskolin (10 μM and 30 μM, for 10 min) or 8-Br-cAMP (100 μM, for 30 min) following protocols previously reported.^53,65^ We show that 8-Br-cAMP and forskolin enhance Ca^2+^ clearance in secretory and maturation stage ameloblasts (Figure 5A–C and Figure S4). Also, to determine if potentiating PMCA affected SOCE, we stimulated SOCE using thapsigargin and show that forskolin does not enhance SOCE in either cell type, only affecting the kinetics of Ca^2+^ clearance (Figure 5D–F).

## 4 | DISCUSSION

Ca^2+^ transport in ameloblasts follows primarily a transcellular route thus requiring net Ca^2+^ influx across the basal/proximal cell membrane, and net efflux across the distal/apical membrane.^2,18,66^ Our recent functional studies indicate that SOCE is the main Ca^2+^ influx mechanism in ameloblasts.^25,38^ These studies are supported by reports of enamel hypomineralization and other enamel defects in patients with dysfunctional SOCE.^60,67,68,69^ Efflux mechanisms, however, are less clear because of a dearth of functional studies.^11,18^ In addition, the subcellular localization in ameloblasts of the various PMCAs have previously resulted in conflicting data hindering a clear picture on the functions of these Ca^2+^ pumps during enamel formation.^12,66^ More recently, the Paine laboratory addressed this question reporting that PMCA1 and PMCA4 are the most abundant isoforms, being more highly expressed in the secretory cells, and both localize to the basolateral pole of the ameloblasts.^37^

The role of Ca^2+^ pumps in enamel formation should consider the differences in function between secretory and maturation stages and how global Ca^2+^ handling differs across stages.^2,16^ Ca^2+^ transport in maturation increases significantly compared to the levels reported in the secretory ameloblasts, a finding in line with the abrupt increase in crystal expansion in maturation.^2,16,24^ PMCAs have been considered important means to deliver Ca^2+^ to the extracellular space^66^ and at least one study showed that perfusing rats with vanadate, a PMCA blocker, decreased the passage of radiolabeled Ca^2+^ (^45^Ca) to the enamel layer, interestingly, associated with maturation stage ameloblasts.^70^ Here, we aimed to provide a functional analysis of the role of PMCAs in ameloblasts Ca^2+^ efflux during the secretory and maturation stages.

Gene expression analysis shown here supports previous data.^37^ PMCA1 and PMCA3 are more highly expressed in the secretory stage ameloblasts (Figure 1A). Consistent with this expression pattern, we show that one of the main PMCA activators, *PKA*, is also more abundantly expressed in the secretory ameloblasts (Figure 1B). Interestingly, the expression levels of calmodulin, which activates PMCAs by Ca^2+^/calmodulin binding, is equally abundant in secretory and maturation stage ameloblasts.^71^

Previous research considered PMCAs as the driving force to extrude Ca^2+^ in secretory ameloblasts.^72^ Overall, our data support the involvement of PMCAs, but we show that this process is differentially affected by the levels of cytosolic Ca^2+^. We found that stimulating secretory and maturation stage ameloblasts with low ATP concentrations to generate a low or a moderate increase in _c_Ca^2+^ evoked a modest but significantly faster Ca^2+^ clearance in secretory relative to maturation ameloblasts (Figure 2). We suggest that this Ca^2+^clearance is PMCA-mediated because blocking the pumps using an alkaline (pH 9.0) perfusate completely blocked Ca^2+^ extrusion in both cell types (Figure 2). Similarly, evoking modest elevations in _c_Ca^2+^ by passively depleting the ER Ca^2+^ stores with thapsigargin shows that secretory ameloblasts are clearing Ca^2+^ faster than maturation cells (Figure 3). Using vanadate or caloxin 1b1 at concentrations reported in the literature^46,48,50,51^ to inhibit PMCAs, we show a significant delay in Ca^2+^ clearance in both ameloblast cell types, with a more prominent effect in secretory ameloblasts, supporting the notion that PMCAs mediate the clearance in both cells but with a higher clearance capacity in secretory ameloblasts (Figure 3).

A different picture develops when larger _c_Ca^2+^ levels are induced following the activation of SOCE. This is likely because at higher concentrations, in addition to PMCAs, the Na^+^/Ca^2+^ exchangers (NCXs/NCKXs) are at work.^29^ We show that SOCE stimulation elicited a significantly higher increase in _c_Ca^2+^ in maturation ameloblasts relative to secretory cells (Figure 4, Figure S3), validating our previous results.^25,38,61^ We also identified faster Ca^2+^ clearance rates in maturation compared to secretory cells, likely the combined result of the activity of both PMCA and the Ca^2+^ exchangers (Figure 4A–F). This is confirmed by the application of an alkaline pH (9.0) medium, which, by contrast with the previous experiments in low Ca^2+^ stimulus, does not effectively block Ca^2+^ extrusion, only delays this process (Figure 4A–C). This suggests that PMCAs are actively extruding Ca^2+^ during maturation despite their gene expression decreasing at that stage. These results also suggest that NCXs/NCKXs contribute to clearance when higher _c_Ca^2+^ elevations are evoked.

Preincubation of ameloblasts with vanadate shows similar trends in delaying Ca^2+^ clearance in both secretory and maturation stage (Figure 4D–F). Previously, it was reported that PMCA4 associated with the ORAI1 channel via partner of STIM1 (POST) in Jurkat-T cells to affect downstream processes modulated by SOCE,^62^ pointing to a PMCA-SOCE connection. Our study further strengthens this connection showing that inhibition of PMCAs using vanadate significantly decreases (~40%) the rate of Ca^2+^ influx via SOCE, but only in secretory ameloblasts (Figure 4D). Histologically, secretory and maturation ameloblasts differ in the subcellular arrangement of intracellular organelles (ER, mitochondria),^73^ which are important in sustaining SOCE. Studies in immune cells revealed both a functional crosstalk between SOCE components (STIM1/ORAI1) and PMCA-mediated Ca^2+^ clearance.^74–76^ Local _c_Ca^2+^ microdomains near the ORAI channels promoted the activity of PMCAs suggesting that they may play an important role in amplitude and spatiotemporal dynamics of Ca^2+^ signals.^75^ Similarly, Barak et al. showed that PMCA4b activity prevented a prolonged _c_Ca^2+^ signal mediated by the ORAI channels.^76^ Thus, a speculative argument would suggest that SOCE requires PMCA-mediated Ca^2+^ clearance to prevent the excessive accumulation of Ca^2+^ near the open ORAI1 channels preventing Ca^2+^ inactivation of ORAI1 in secretory ameloblasts, but this needs to be addressed in detail in future studies. Regardless, this is a novel role of PMCA in ameloblast Ca^2+^ handling linked to SOCE.

It has been documented that the phosphorylation of PMCA enhances the function of these pumps.^32,63,77^ PKA phosphorylates PMCA and *PKA* transcripts are abundant in enamel cells, particularly in the secretory stage (Figure 1). Activation of the PKA signaling pathway is mediated by an increase in the levels of cAMP in response to adenyl cyclase stimulation.^78^ Interestingly, the expression of cAMP has been reported in enamel cells but its role in amelogenesis is yet to be determined.^79^ Although the analysis of PKA signaling in enamel cells is beyond the scope of this work, our primary interest focused on the possibility that cAMP and PKA could be involved in Ca^2+^ clearance by PMCA. Our results show that potentiating PMCA function with 8-Br-cAMP or forskolin, both acting via the cAMP-PKA axis, enhanced Ca^2+^ clearance (Figure 5A,B). Interestingly, forskolin stimulation of secretory and maturation enamel cells did not affect the kinetics of SOCE (Figure 5D,E).

The results shown above indicate that PMCAs are functionally involved in the removal of _c_Ca^2+^ in both secretory and maturation stage ameloblasts. The degree of this involvement is associated with the magnitude of the _c_Ca^2+^ elevation. PMCAs of secretory ameloblasts are faster than maturation stage cells at clearing low and/or moderate volumes of _c_Ca^2+^, and these data also suggest that in the secretory stage, PMCAs are the main system in the extrusion of _c_Ca^2+^. At higher _c_Ca^2+^elevations, for example when SOCE is activated, PMCAs continue to be active components of the Ca^2+^ extrusion system, but in this instance, it is likely the Na^+^/Ca^2+^ exchangers are the primary Ca movers, an issue that requires further analysis.

The functional studies shown here emphasize that PMCAs are not merely housekeeping proteins controlling basal levels of _c_Ca^2+^ levels, rather they play an active role in Ca^2+^ extrusion in the ameloblasts of both stages and, therefore, are important contributors to enamel mineralization. This is supported by a previous report showing deficient levels of Ca^2+^ in the enamel of rats treated with vanadate.^70^ In summary, we have provided a functional analysis of PMCAs in ameloblast Ca^2+^ extrusion. However, this important system is not operating in isolation during amelogenesis, the more efficient NCX/NCKX proteins are also expressed in the ameloblasts and would likely be functionally active. Disentangling the unique roles of PMCAs and Na^+^/Ca^2+^ exchangers in amelogenesis is warranted and will provide valuable information to understand the unique composition of the Ca^2+^ clearance system in these cells and how it may impact the development of enamel mineralization in physiology and disease.

## Supplementary Material

Suppl Mat

## Data Availability

The data that support the findings of this study are available in the methods and/or supplementary material of this article.

## Abbreviations:

8-Br-cAMP: 8- Bromoadenosine- 3′,5′-cyclic monophosphate
ATP: adenosine triphosphate
cCa^2+^: cytosolic Ca^2+^
FSK: forskolin
MAT: maturation
NCX: sodium/calcium exchanger
NCKX: sodium/potassium/calcium exchanger
NMDG: *N*-Methyl-D-glucamine
PKA: protein kinase A
PMCA: plasma membrane Ca^2+^ ATPase
SEC: secretory
SOCE: store operated Ca^2+^ entry
STIM1: stromal interaction molecule 1
VAN: vanadate
