# Supplementary material for: PMCA Ca2+ clearance in dental enamel cells depends on the magnitude of cytosolic Ca2+
Source: FASEB J. 2022 Dec 14;37(1):e22679. doi: 10.1096/fj.202201291R (PMC11006021; doi:10.1096/fj.202201291R)
Supplement: Supplementary file 1 — Appendix S1: [file FSB2-37-e22679-s001.pdf]

Supplementary Materials for

PMCA Ca^{2+} clearance in dental enamel cells depends on the magnitude of cytosolic Ca^{2+}

Guilherme Henrique Souza Bomfim[1], Marta Giacomello[2,3], Rodrigo S. Lacruz[1*]

[1]Department of Molecular Pathobiology, New York University College of Dentistry, NY 10010, USA.
[2,3]Department of Biology, University of Padova, Padua, Italy, Department of Biomedical Sciences, University of Padova, Padua, Italy.

Souza Bomfim, GH. ORCID: https://orcid.org/0000-0001-9454-7266

Lacruz, RS. ORCID: https://orcid.org/0000-0002-0776-6143

* Corresponding author: Rodrigo S. Lacruz; Department of Molecular Pathobiology, New York University College of Dentistry, New York, NY 10010, USA.

E-mail address: rsl10@nyu.edu

Fig. S1. Purity of the enamel ameloblasts culture confirmed using a PE-conjugated monoclonal anti-rat CD90 antibody and *Enam* and *Odam* expression by RT-PCR in secretory (SEC) and maturation (MAT) ameloblasts.

Fig. S2. Comparison between $_{c}Ca^{2+}$ levels evoked by ATP and SOCE.

Fig. S32. Ringer's solution replacement does not affect $_{c}Ca^{2+}$ concentrations in ameloblasts.

Fig. S4. Forskolin 10 μM potentiate PMCA Ca^{2+} clearance in enamel cells.

Table S1. Rat primer sequences used for qRT-PCR.

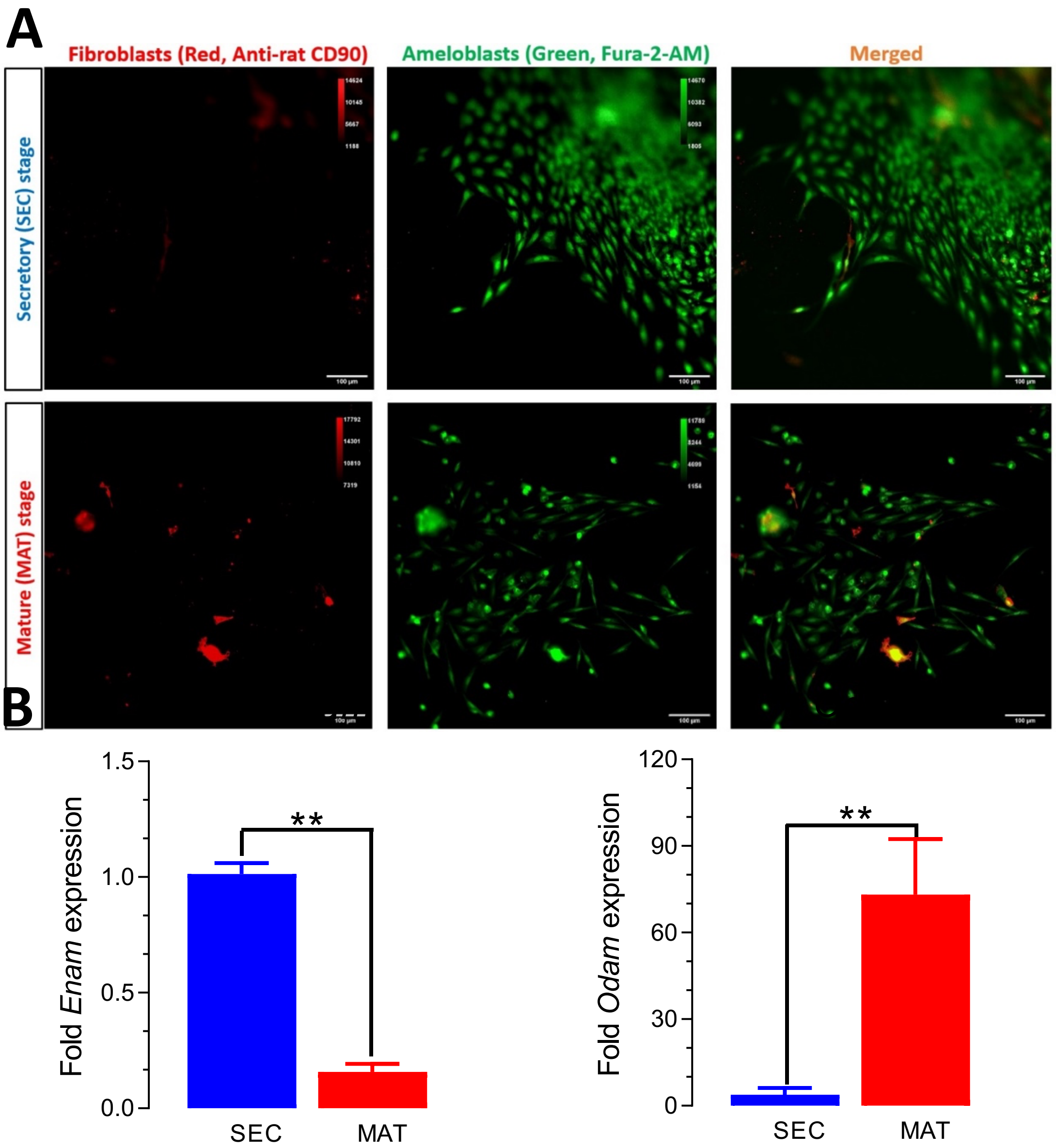

Fig. S1. Confirmation of the purity of the enamel organ cell dissections. (A) Representative fluorescence images of primary ameloblast cultures labelled using a CD90 antibody for fibroblasts (red left panels) or loaded with the ratiometric Ca^{2+} probe Fura-2-AM (green center panels). Red and green images were merged (right panels) and the overlapping cells were not included in the analysis. Scale bars: 100 µm. **(B)** Purity of the ameloblasts culture was confirmed by gene markers *Enam* (enamelin) and *Odam* (odontogenic ameloblast-associated protein) expression in secretory (SEC) and maturation (MAT) stage ameloblasts, respectively, by RT-PCR. Data represent the mean ± SEM of minimum 4 independent experiments. Data analyzed by two-tailed unpaired Student's t-test at $^{**}P < 0.01$ vs. secretory (SEC) group.

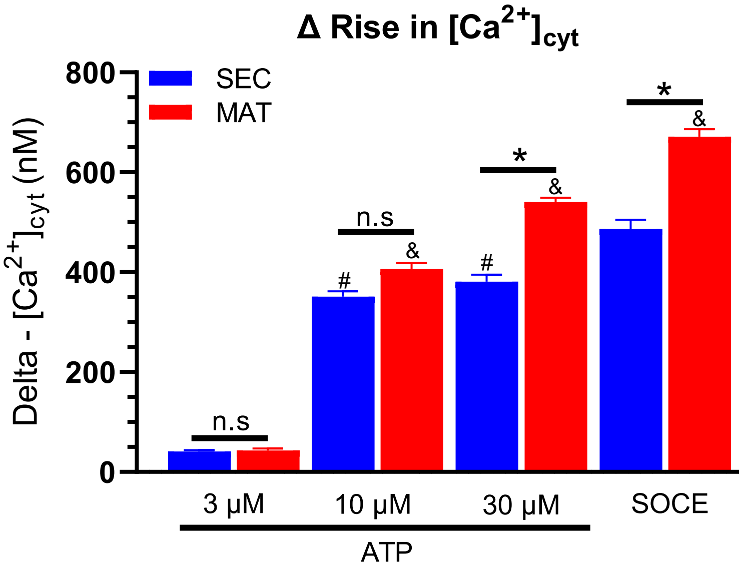

Fig. S2. Comparison between $_{c}Ca^{2+}$ levels evoked by ATP and SOCE. A rise in $_{c}Ca^{2+}$elicited by ATP (3 µM, 10 µM and 30 µM) in secretory (SEC) and maturation (MAT) ameloblast stages compared to levels evoked by SOCE stimulation. Histograms show the delta (∆) Ca^{2+} peak. Data represent the mean ± SEM of ≥40 cells from 3-5 independent experiments analyzed by one-way ANOVA followed by Tukey's multiple comparison post-hoc test. *P < 0.05 vs. comparing maturation relative to secretory stage under the same conditions. #P < 0.05 and denotes comparison between SEC stage cells from 3 µM and 10 µM ATP, between 10 µM and 30 µM ATP. &P < 0.05 denotes comparisons across MAT for subsequent treatments (ATP 3 µM vs 10 µM, 10 µM vs 30 µM, and 30µM ATP vs SOCE). n.s., non-significant.

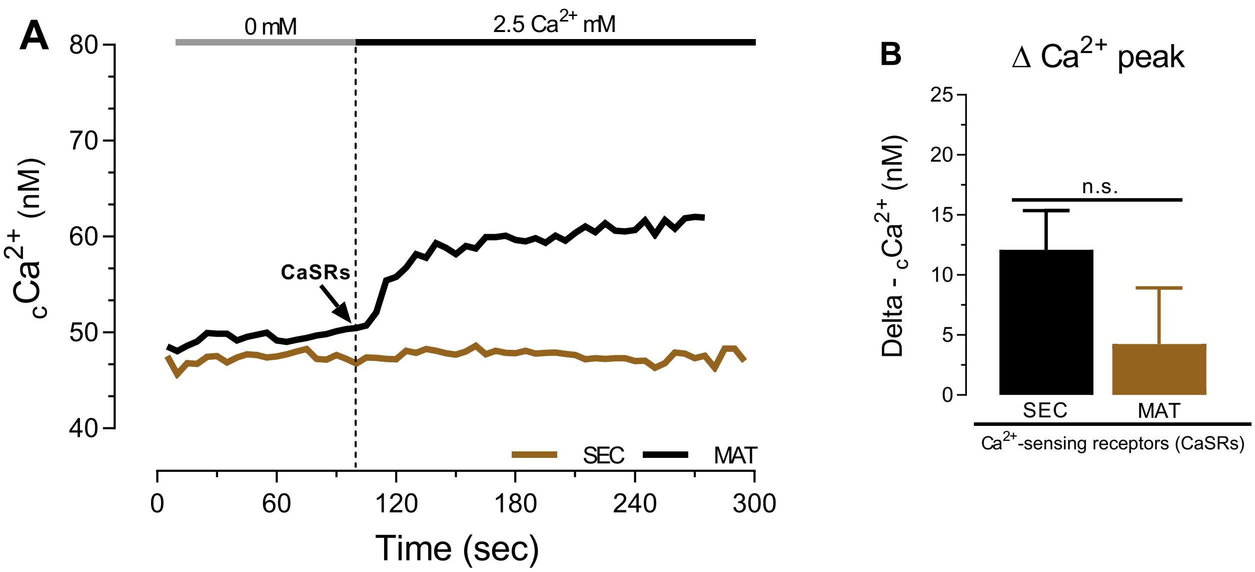

Fig. S3. Ringer's solution replacement does not affect $_cCa^{2+}$ concentrations in ameloblasts. (A) Changes in external solutions with or without Ca^{2+} does not alter $_cCa^{2+}$ in secretory (SEC) or maturation (MAT) stage ameloblasts. We evaluated $_cCa^{2+}$ levels in Ca^{2+}-free Ringer's solution followed by a re-addition of 2.5 mM of extracellular Ca^{2+} in normal Ringer's. **(B)** Delta (Δ) Ca^{2+} peak. Data represent the mean ± SEM of 3 independent experiments analyzed by two-tailed unpaired Student's t-test at n.s., non-significant.

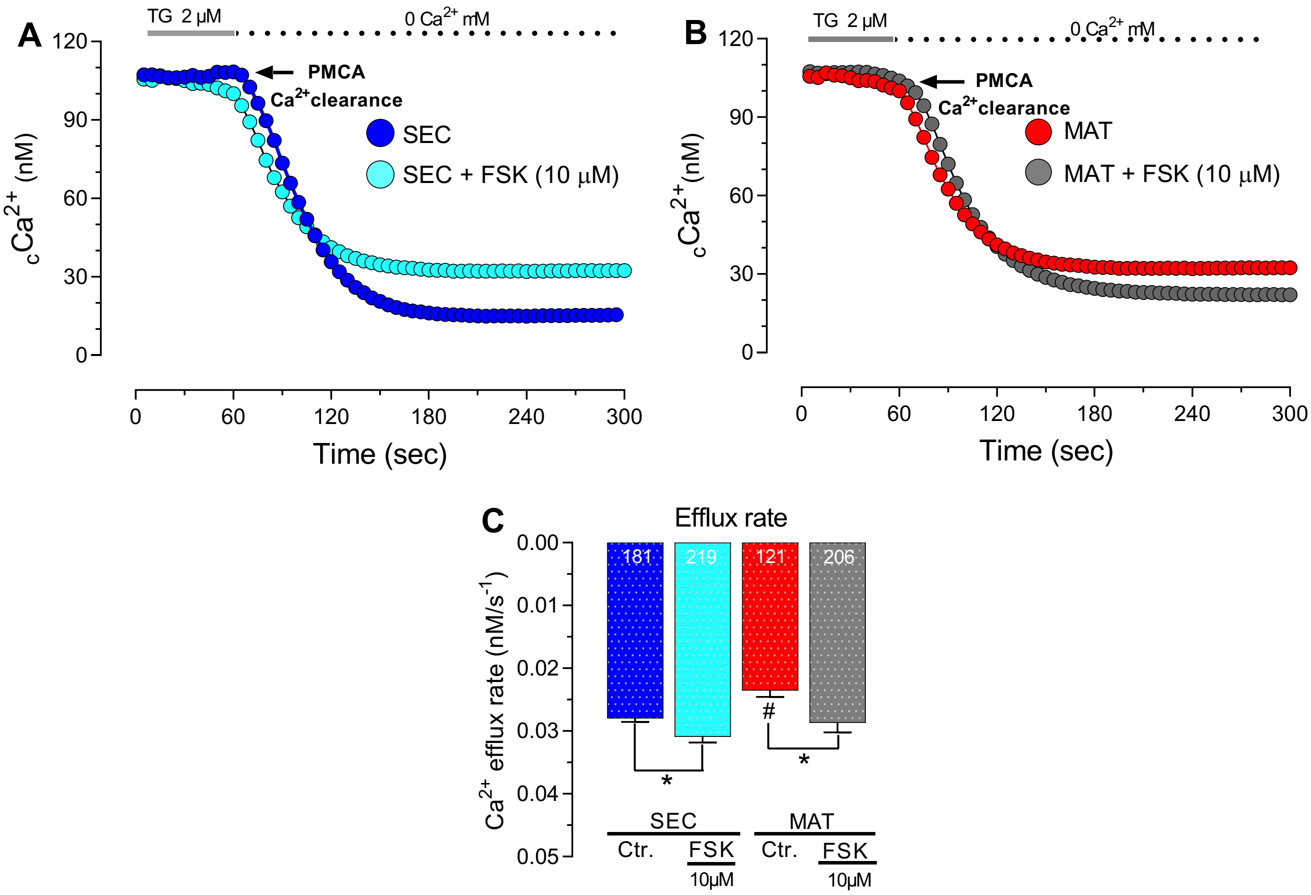

Figure S4. Low forskolin stimulation potentiates PMCA Ca^{2+} clearance in enamel cells. Representative original traces of secretory (SEC) **(A)** and maturation (MAT) **(B)** ameloblasts showing $_{c}Ca^{2+}$ transients recorded after pre-incubation with thapsigargin (TG,15 min, 2 µM) followed by perfusion of Ca^{2+}-free Ringer's (60 to 300s). Ca^{2+} clearance via PMCAs was monitored in the absence or presence of forskolin (10 µM, for 10 min). Quantification of the rate of Ca^{2+} efflux is shown in **(C)**. Data represent the mean ± SEM of ≥121 cells from 3-5 independent experiments analyzed by one-way ANOVA followed by Tukey's multiple comparison post-hoc test. *$P < 0.05$ vs. #$p < 0.05$ (#denotes differences between SEC and MAT).n.s. non-significant.

Table S1. Rat primers sequences used for qRT-PCR.

Table 1. List of Rat *(Rattus norvegicus)* primers couples used

Gene	Forward sequence (5' → 3')	Reverse sequence (3' → 5')	Product Length
Actb (Actin-β)	CACACTGTGCCCATCTATGA	CCGATAGTGATGACCTGACC	272
Atp2b1 (PMCA1)	AAAGCAGGTCTGCTGATGTC	GACGGAGTAAGCCAGTGAGA	219
Atp2b3 (PMCA3)	GCTCCATGACGTAACCAATC	GCGGAATATTGTGGGTGTAG	207
Atp2b4 (PMCA4)	AATCCAAGAACCAGGTCTCC	ACGGCATTGTTATTCGTGTT	184
Odam	TTGCAGCTTTGTAGGCACA	TGACCTTCTGTTCTGGAAGC	206
Enam	TATGGTCTTCCACCAAGGAA	TAGGCACACCATCTCCAAAT	181
Prkaca (PKA-α)	AGAGTGAATCGGACTCGGACG	GCCACGGTTTGCATACTGACC	383
Prkacb (PKA-β)	TAAACCGGTTCACAAGGCGTG	GTTACCAACGCATCTTCCAAC	326
Prkca (PKC-α)	TTTGTTACTTTCTCTTGTCCGGGT	ACATTCATGTCGCAGGTGTCGCA	176
Prkcg (PKC-γ)	TTGATGGGGAAGATGAGGAGG	GAAATCAGCTTGGTCGATGCTG	347
