# Supplementary material for: PMCA Ca2+ clearance in dental enamel cells depends on the magnitude of cytosolic Ca2+
Source: FASEB J. Author manuscript; Available in PMC 2024 Apr 10. (PMC11006021; doi:10.1096/fj.202201291R)
Supplement: Supplementary file 1 [file NIHMS1980723-supplement-Suppl_Mat.pdf]

Supplementary Materials for

PMCA Ca^{2+} clearance in dental enamel cells depends on the magnitude of cytosolic Ca^{2+}

Guilherme Henrique Souza Bomfim[1], Marta Giacomello[2,3], Rodrigo S. Lacruz[1*]

[1]Department of Molecular Pathobiology, New York University College of Dentistry, NY 10010, USA.
[2,3]Department of Biology, University of Padova, Padua, Italy, Department of Biomedical Sciences, University of Padova, Padua, Italy.

Souza Bomfim, GH. ORCID: https://orcid.org/0000-0001-9454-7266

Lacruz, RS. ORCID: https://orcid.org/0000-0002-0776-6143

* Corresponding author: Rodrigo S. Lacruz; Department of Molecular Pathobiology, New York University College of Dentistry, New York, NY 10010, USA.

E-mail address: rsl10@nyu.edu

Fig. S1. Purity of the enamel ameloblasts culture confirmed using a PE-conjugated monoclonal anti-rat CD90 antibody and *Enam* and *Odam* expression by RT-PCR in secretory (SEC) and maturation (MAT) ameloblasts.

Fig. S2. Comparison between $_{c}Ca^{2+}$ levels evoked by ATP and SOCE.

Fig. S3. Ringer's solution replacement does not affect $_{c}Ca^{2+}$ concentrations in ameloblasts.

Fig. S4. Forskolin 10 µM potentiate PMCA Ca^{2+} clearance in enamel cells.

Table S1. Rat primer sequences used for qRT-PCR.

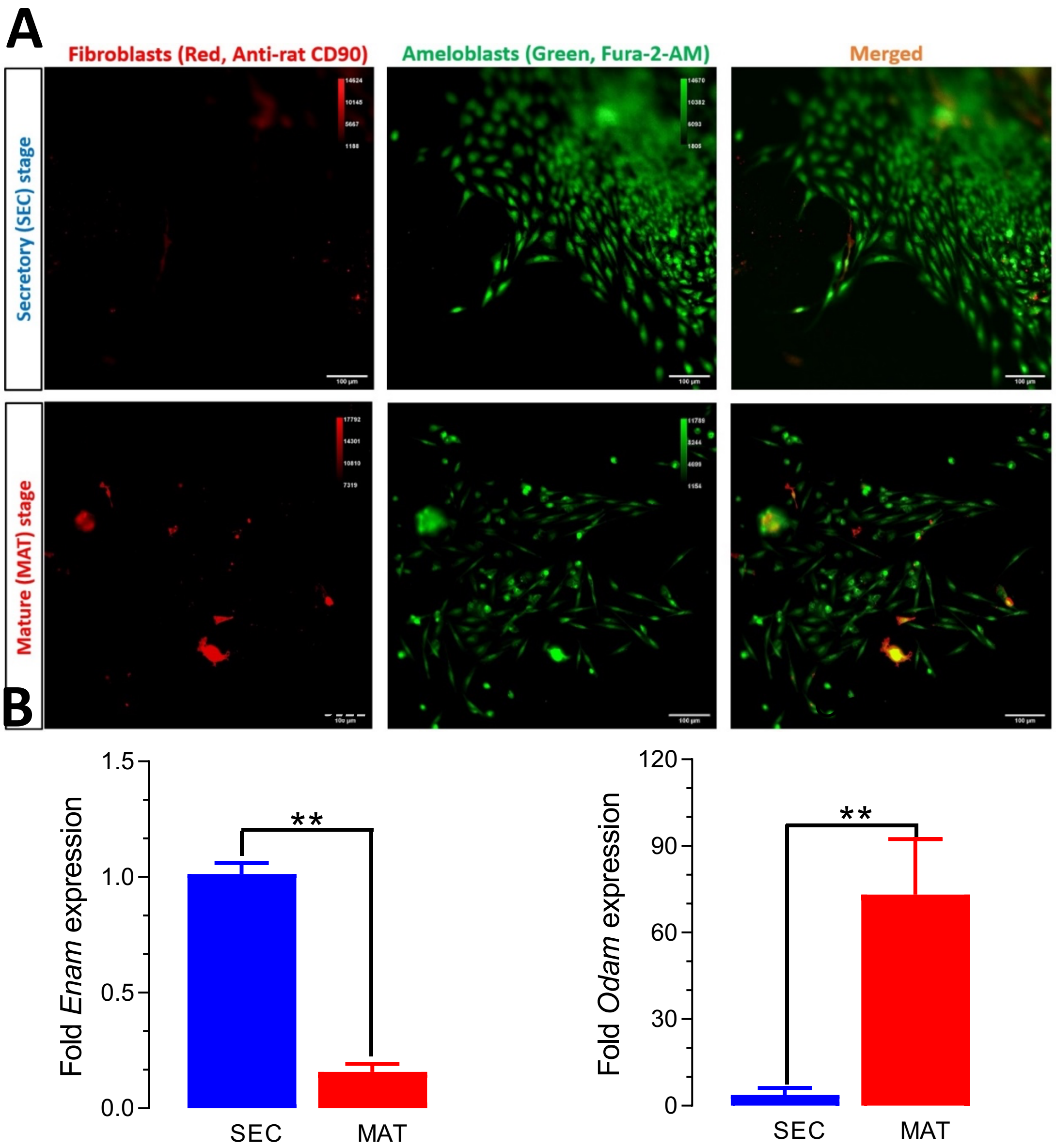

Fig. S1. Confirmation of the purity of the enamel organ cell dissections. (A) Representative fluorescence images of primary ameloblast cultures labelled using a CD90 antibody for fibroblasts (red left panels) or loaded with the ratiometric Ca^{2+} probe Fura-2-AM (green center panels). Red and green images were merged (right panels) and the overlapping cells were not included in the analysis. Scale bars: 100 µm. **(B)** Purity of the ameloblasts culture was confirmed by gene markers *Enam* (enamelin) and *Odam* (odontogenic ameloblast-associated protein) expression in secretory (SEC) and maturation (MAT) stage ameloblasts, respectively, by RT-PCR. Data represent the mean ± SEM of minimum 4 independent experiments. Data analyzed by two-tailed unpaired Student's t-test at $^{**}P < 0.01$ vs. secretory (SEC) group.

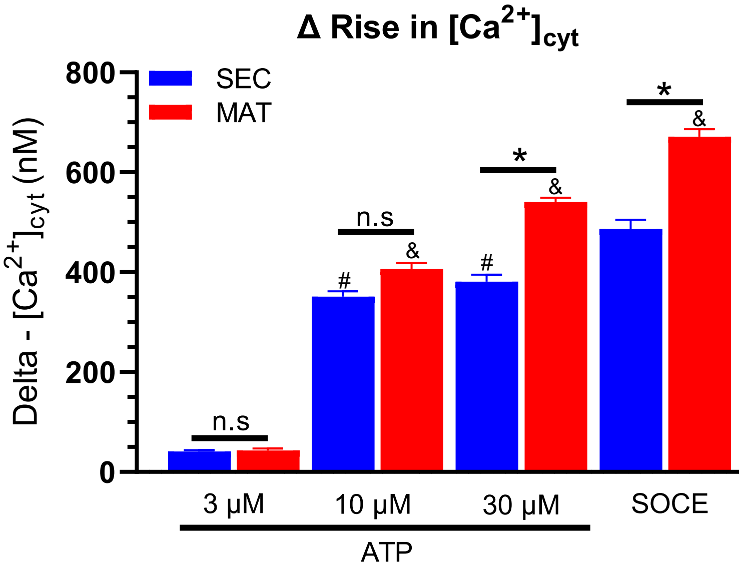

Fig. S2. Comparison between $_{c}Ca^{2+}$ levels evoked by ATP and SOCE. A rise in $_{c}Ca^{2+}$elicited by ATP (3 µM, 10 µM and 30 µM) in secretory (SEC) and maturation (MAT) ameloblast stages compared to levels evoked by SOCE stimulation. Histograms show the delta (Δ) Ca^{2+} peak. Data represent the mean ± SEM of ≥40 cells from 3-5 independent experiments analyzed by one-way ANOVA followed by Tukey's multiple comparison post-hoc test. *P < 0.05 vs. comparing maturation relative to secretory stage under the same conditions. #P < 0.05 and denotes comparison between SEC stage cells from 3 µM and 10 µM ATP, between 10 µM and 30 µM ATP. &P < 0.05 denotes comparisons across MAT for subsequent treatments (ATP 3 µM vs 10 µM, 10 µM vs 30 µM, and 30µM ATP vs SOCE). n.s., non-significant.

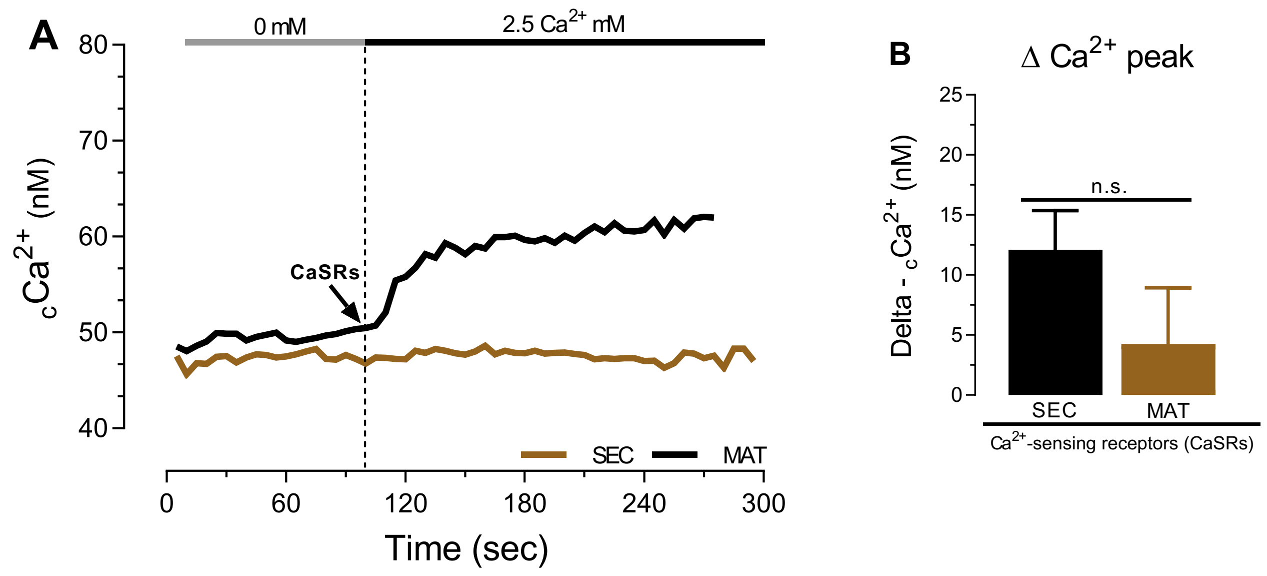

Fig. S3. Ringer's solution replacement does not affect $_{c}Ca^{2+}$ concentrations in ameloblasts. **(A)** Changes in external solutions with or without Ca^{2+} does not alter $_{c}Ca^{2+}$ in secretory (SEC) or maturation (MAT) stage ameloblasts. We evaluated $_{c}Ca^{2+}$ levels in Ca^{2+}-free Ringer's solution followed by a re-addition of 2.5 mM of extracellular Ca^{2+} in normal Ringer's. **(B)** Delta (Δ) Ca^{2+} peak. Data represent the mean ± SEM of 3 independent experiments analyzed by two-tailed unpaired Student's t-test at n.s., non-significant.

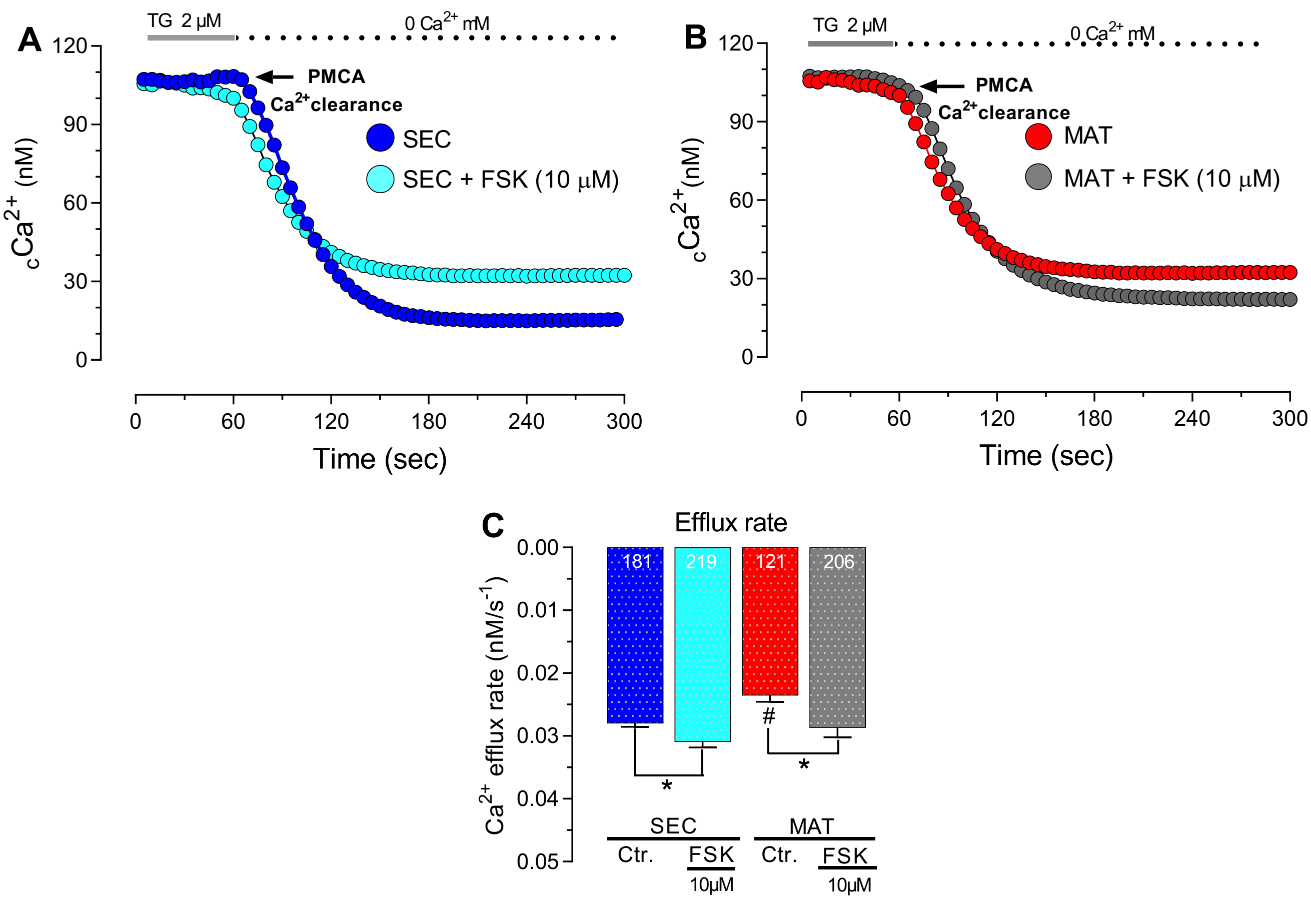

Figure S4. Low forskolin stimulation potentiates PMCA Ca^{2+} clearance in enamel cells. Representative original traces of secretory (SEC) **(A)** and maturation (MAT) **(B)** ameloblasts showing $_{c}Ca^{2+}$ transients recorded after pre-incubation with thapsigargin (TG,15 min, 2 µM) followed by perfusion of Ca^{2+}-free Ringer's (60 to 300s). Ca^{2+} clearance via PMCAs was monitored in the absence or presence of forskolin (10 µM, for 10 min). Quantification of the rate of Ca^{2+} efflux is shown in **(C)**. Data represent the mean ± SEM of ≥121 cells from 3-5 independent experiments analyzed by one-way ANOVA followed by Tukey's multiple comparison post-hoc test. *P < 0.05 vs. #p < 0.05 (#denotes differences between SEC and MAT).n.s. non-significant.

Table S1. Rat primers sequences used for qRT-PCR.

Table 1. List of Rat *(Rattus norvegicus)* primers couples used

Gene	Forward sequence (5' → 3')	Reverse sequence (3' → 5')	Product Length
Actb (Actin-β)	CACACTGTGCCCATCTATGA	CCGATAGTGATGACCTGACC	272
Atp2b1 (PMCA1)	AAAGCAGGTCTGCTGATGTC	GACGGAGTAAGCCAGTGAGA	219
Atp2b3 (PMCA3)	GCTCCATGACGTAACCAATC	GCGGAATATTGTGGGTGTAG	207
Atp2b4 (PMCA4)	AATCCAAGAACCAGGTCTCC	ACGGCATTGTTATTCGTGTT	184
Odam	TTGCAGCTTTGTAGGCACA	TGACCTTCTGTTCTGGAAGC	206
Enam	TATGGTCTTCCACCAAGGAA	TAGGCACACCATCTCCAAAT	181
Prkaca (PKA-α)	AGAGTGAATCGGACTCGGACG	GCCACGGTTTGCATACTGACC	383
Prkacb (PKA-β)	TAAACCGGTTCACAAGGCGTG	GTTACCAACGCATCTTCCAAC	326
Prkca (PKC-α)	TTTGTTACTTTCTCTTGTCCGGGT	ACATTCATGTCGCAGGTGTCGCA	176
Prkcg (PKC-γ)	TTGATGGGGAAGATGAGGAGG	GAAATCAGCTTGGTCGATGCTG	347
